# Shell dissolution rates differ fourfold between mussel species

**DOI:** 10.1098/rsos.250664

**Published:** 2025-07-16

**Authors:** Rachel R. Carlson, Mazie A. Lewis, Aaron T. Ninokawa, Alisha M. Saley, Tessa M. Hill, Brian Gaylord

**Affiliations:** ^1^ Bodega Marine Laboratory, University of California Davis, Bodega Bay, CA, USA; ^2^ Department of Environmental Science, Policy, and Management, University of California Berkeley Rausser College of Natural Resources, Berkeley, CA, USA; ^3^ Department of Earth and Planetary Sciences, University of California Davis, Davis, CA, USA; ^4^ Chemistry, SUNY College of Environmental Science and Forestry, Syracuse, NY, USA; ^5^ Department of Evolution and Ecology, University of California, Davis, Davis, CA, USA

**Keywords:** ocean acidification, climate change, mussel, coastal, calcification, freshwater

## Abstract

Ocean acidification poses a critical threat to marine calcifiers globally and is particularly severe in the California Current System, where ecologically and economically important bivalves experience reduced calcification under climate change. Marine mussels display differential habitat preferences, with species like *Mytilus californianus* favouring fully saline environments and *M. trossulus* inhabiting sites with greater freshwater input. Determining abiotic dissolution rates of these species under ocean acidification is essential for predicting future consequences of climate change for coastal populations. We examined shell dissolution rates of mussel congeners under a range of pH (6.5–9.3) and aragonite saturation states (0.1–9.0). We also experimentally quantified the relative importance of dissolution from interior versus exterior shell surfaces. *M. trossulus* exhibited fourfold higher shell dissolution rates than *M. californianus*. When the shell interior was sealed against seawater exposure, dissolution rates decreased significantly in both species, indicating high abiotic dissolution on the shell interior. Results demonstrate that dissolution rates can vary between congeners inhabiting the same biogeographic region. Our finding that freshwater-tolerant *M. trossulus* has higher abiotic dissolution under ocean acidification is important because low salinity may further retard calcification, altering future intertidal population structure along freshwater-influenced coastlines.

## 1. Introduction

Ocean acidification is increasing worldwide due to elevated carbon dioxide in the atmosphere, leading to modifications in the ocean carbonate system and, consequently, alterations in the shell structure of calcifying organisms like marine mussels [1–3]. Ocean acidification is particularly severe in the California Current of the northeast Pacific Ocean, where pH is decreasing twice as rapidly as the global average [4–6] and nearshore aragonite saturation state (Ω_aragonite_) is expected to move outside of its current variability envelope by the mid−2030s [7]. Significant changes to calcifying organisms have been documented due to ocean acidification in this region including impaired calcification (e.g. mussel shell thinning [8–10]), modified larval development and reproduction (e.g. [11,12]) and altered behaviour or susceptibility to predation (e.g. [13–15]) that could disrupt species assemblages and trophic structure in critical fisheries [16–19].

Mussels are important habitat-forming organisms that are highly susceptible to ocean acidification. Mussels generate rugose beds that increase benthic surface area in the rocky intertidal zone, forming climatic niches for epibionts and small benthic organisms [20] and support aquaculture and coastal fisheries (e.g. [21,22]). Many mussel species in the California Current are vulnerable to ocean acidification, posing serious risks to benthic ecosystems [8,23–25]. Two congener mussels, *M. trossulus* and *M. californianus*, are common in the California Current yet inhabit divergent habitat niches. *M. trossulus* is abundant in the Pacific Northwest but rare in California and thrives in regions characterized by high freshwater exposure such as the Puget Sound, and is likewise abundant in the Baltic Sea [26]. In contrast, *M. californianus* spans from northern Mexico to Alaska and prefers typical marine salinity levels of 32−37 ppt [10,21]. Given that fresh water sources vary in alkalinity, which alters the relationship between dissolved carbon dioxide and pH, *M. trossulus* and *M. californianus* may experience differential susceptibility to dissolution when their respective habitats are subjected to increased carbon dioxide.

Phenotypic differences between *M. trossulus* and *M. californianus* may also affect vulnerability to ocean acidification. *M. californianus* was recently characterized as the only Mytilid species to precipitate three mineralogical layers—outer calcite, middle aragonite and inner calcite—whereas other *Mytilus* species precipitate two layers: an outer layer of prismatic calcite and an inner layer of nacreous aragonite [10]. Inner-shell mineralogy may strongly affect vulnerability to ocean acidification, as the inner shell of mussels may be more susceptible to high pCO_2_ than the outer shell due to the fact that the inner shell lacks a periostracum (protective outer layer [27]). While the outer shell and periostracum are exposed to seawater, the inner shell is exposed to extrapallial fluid that has reduced pH and carbonate, associated with diffusive ejection of metabolic pCO_2_ by mussels that exacerbates acidified conditions at the inner shell compared to seawater [28,29]. Fitzer *et al.* [30] found that, under conditions of ocean acidification, calcite outer shells in *M. edulis* became more brittle, while aragonite inner shells became softer and more pliable. Melzner *et al.* [27] found that *M. edulis*, which is similar in morphology and habitat to *M. trossulus*, exhibited high internal shell dissolution under elevated seawater pCO_2_ and low-food treatments, though food subsidies helped mussels overcome high pCO_2_ stress. However, no study to our knowledge has directly compared the effects of ocean acidification on mussel shells with differential shell structure.

In this study, we seek to address the following research questions. First, how do abiotic dissolution rates differ between taxonomically similar calcifiers (congeners) with different mineralogical composition (*M. trossulus* and *M. californianus*)? Second, if differences in abiotic dissolution are observed between congeners, to what extent does the shell interior versus exterior contribute to this difference (i.e. which shell surface introduces heightened vulnerability)? (Note: throughout this article, we use ‘abiotic dissolution’ to describe shell loss independent of the biological activity of live mussels or shell-associated microbes.) We discuss possible implications of results for organism vulnerability to ocean acidification. We also contextualize results in the divergent environments (levels of fresh water input) these organisms typically inhabit.

## 2. Methods

### 2.1. Mussel collection and preparation

Naturally settled *M. californianus* were manually collected from Carmet Beach, CA, USA between January 2020 and April 2022 [31]. Salinities at a monitoring station in the vicinity of Carmet Beach exhibit a mean of 33.2 ± 0.4 standard deviation [32]. Mussels were hand-selected at lengths ranging from 30 to 80 mm from the mid-intertidal zone and samples were gathered from the same location to draw from a consistent population and set of environmental parameters that can determine phenotypic traits. Mussels were dissected immediately upon arrival at Bodega Marine Laboratory (BML), CA (<0.5 h transit time) to remove all body tissue. Remaining organic materials (byssal threads, epibionts) were removed by drying shells in an oven for 24 h at 60°C.

*M. trossulus* of 20–75 mm length (target length consistent with *M. californianus* shells) were collected from Penn Cove Shellfish Farm in Penn Cove, WA, USA in September and December of 2023. Penn Cove, which uses natural seed, catching and growing wild larvae on rope substrate suspended from rafts, is located in the Salish Sea, which receives significant fresh water from the Skagit River and other waterways in British Columbia and the Pacific Northwest including the Whidbey Basin watershed. Monitoring data at the farm shows a mean autumn salinity of 21.1 ± 5.3 and mean pH of 7.79 ± 0.24 at 1 m depth [33]. Mussels from Penn Cove were maintained in a moist, cool and insulated environment during shipment to BML (< 1 day), and were dissected immediately upon arrival at the lab using methods described above.

To separate contributions of the inner or outer shell surface to dissolution, we coated the inside of a subset of shells of each species with a clear, silicone waterproof sealant (Loctite), applying a single, thin layer to the nacre. A total of 49 *M*. *trossulus* shells were sealed and 73 were unsealed; and 74 *M*. *californianus* shells were sealed and 46 were unsealed. Sealed *M. californianus* shells were completed by Saley & Gaylord [34,35] but, given that these authors were attempting to sample across a wide range of periostracum percent cover, we used only shells with a comparable percentage of periostracum to other treatment groups (>50% periostracum; 25 of original 74). Periostracum in Saley & Gaylord [35] was determined through digital photographs analysed in ImageJ (software version 1.52a); periostracum in other treatments was estimated >50% based on visual inspection. The sealant was left to dry for >48 h prior to incubation and all shells were then inspected to ensure full seal and soaked in ambient seawater for 1 h immediately prior to incubation (described below) to allow seawater to saturate any possible air pockets in the sealant. Trials conducted in advance demonstrated that cured sealant had no detectable effect on seawater chemistry [35].

### 2.2. Mussel treatment

Abiotic dissolution experiments were conducted on mussel shells between March 2020 and March 2024. All mussel shells were incubated in seawater manipulated to a target aragonite saturation state of Ω_aragonite_ = 0–9 (actual Ω_aragonite_ = 0.05–9.03). Seawater was derived from the Bodega Marine Lab seawater flow-through system, sourced from an intake located 60 m offshore of Horseshoe Cove, CA at a depth of 2 m below mean low tide, and sand filtered to 30 microns. Incubations were conducted in dark conditions at a constant temperature of mean 12.4°C, corresponding to ambient seawater temperature during the season when incubations were conducted. We first added ambient seawater to a 1 l mixing vessel, then added variable doses of sodium hydroxide (NaOH) and hydrochloric acid (HCl) to manipulate pH and Ω_aragonite_. We mixed the treatment water thoroughly and subsampled 150 ml of this water to characterize chemical conditions before incubation (described below). The remaining 850 ml of treatment water was inverted into a glass incubation jar containing a mussel shell and the jar was sealed immediately and placed in a dark incubation chamber. While dark conditions are primarily used to control light conditions in live-mussel experiments, several incubations occurred in tandem with live incubations in darkness for a separate experiment and therefore dark conditions were maintained throughout our experiment for consistency. We removed mussel shells after a target of 42−45 h, though 25% of incubations occurred in tandem with prior experiments and had an incubation period of 108−115 h; we accounted for this difference by normalizing abiotic dissolution by time (dissolution rate). After incubation, a 150 ml subsample was again extracted from jars and used to measure chemical conditions after incubation. The treatment water mass and shell mass were recorded, with water mass derived using the equation (total mass = jar mass + shell mass +water mass).

Before and after incubation, we measured oxygen, temperature, salinity, total alkalinity and pH in each incubation vessel. Ammonia was measured before incubation from one of four carboys used to dispense ambient seawater for treatments (triplicate ammonia samples per carboy) and after incubation from each incubation vessel. Oxygen and temperature were measured using a PreSens Microx 4 micro-optode; salinity with a Horiba Laqua PC 1100 conductivity probe; and pH with a Horiba Laqua PC 1100 instrument, with all four probes simultaneously submerged in a 150 ml subsample. pH measurements were calibrated on the total scale using simultaneous spectrophotometry on a 3 ml sample within our temperature-controlled room using m-cresol purple dye, which is sensitive to pH changes [36]. Before and after incubation, our 150 ml subsamples were preserved in duplicate, using opaque bottles for alkalinity titration, which occurred within 24 h according to methods described in Ninokawa *et al.* [31]. Duplicate samples that displayed a standard deviation >10 were discarded, resulting in 45 unsealed and 27 sealed *M. trossulus* shells and 46 unsealed and 25 sealed *M. californianus* shells used for analysis (table 1). Though waste excretion was not expected for abiotic shells, we measured ammonia to account for any biologically associated changes in alkalinity (i.e. microbial activity in water or on shells) using a salicylate spectrophotometric assay, as described in Ninokawa *et al.* [31]. Finally, we calculated abiotic dissolution rate using the ammonia-corrected alkalinity anomaly technique [37], dividing CaCO_3_ dissolution by incubation time and dry shell mass. We used chemical measurements to determine the carbonate chemistry of each incubation including Ω_aragonite_ and Ω_calcite_ using the package *seacarb* v 3.3.3 in R v 4.3.3 with constants from Lueker *et al.* [38].

**Table 1.** Mussel treatments and summary statistics (mean ± s.d.).

	sample size	collection site	mean autumn salinity at collection site	mean dry weight (g)	mean length (mm)	mean treat. time (h)	maximum dissolution rate (µmol hr^−1^ g^−1^)	dissolution rate at Ω < 1 (µmol h^−1^ g^−1^)
*M. trossulus*—inner shell unsealed	45	Penn Cove, WA, USA	21.1 ± 5.3 [33]	4.1 ± 1.0	58.9 ± 5.2	43.2 ± 0.6	**0.39**	**0.28 ±** **0.07**
*M. trossulus*—inner shell sealed	27	Penn Cove, WA, USA	21.1 ± 5.3 [33]	3.6 ± 0.8	54.1 ± 4.5	43.7 ± 0.8	**0.14**	**0.07 ±** **0.04**
*M. californianus*—inner shell unsealed	46	Carmet, CA, USA	33.2 [32]	14.4 ± 5.4	NA	111.1 ± 2.5	**0.09**	**0.06 ±** **0.02**
*M. californianus*—inner shell sealed	25	Carmet, CA, USA	33.2 [32]	10.5 ± 1.9	54.2 ± 4.2	49.2 ± 2.2	**0.02**	**0.01 ±** **0.0**

Sealed *M. californianus* shells were tested during a separate experiment by Saley & Gaylord [34,35] using the same analytical methods but targeting a narrow range of Ω_aragonite _< 1 and using approximately 300 ml of water. Therefore, in statistical analyses focused on comparisons across groups, our full dataset was subsampled to Ω_aragonite _< 1 for consistency. We also normalized dissolution (in µmol kg^−1^) by water mass, multiplying alkalinity change by kg treatment water before finding dissolution rate per shell mass (µmol CaCO_3_ hr^−1^ g^−1^) across all treatments. Note that shell length but not mass was measured by Saley & Gaylord [35]; we therefore derived shell mass for this group based on the relationship between length and mass in a separate dataset of 558 *M*. *californianus* mussels initially measured for Ninokawa *et al.* [31].

### 2.3. Statistical analysis

To evaluate the difference between abiotic dissolution rate of two congener species, we used bootstrapping to compare dissolution rate per shell mass (µmol hr^−1^ g^−1^) by sampling with replacement from *M. trossulus* and *M. californianus* shell incubations with 10 000 iterations. Bootstrapping allowed us to estimate 95% confidence intervals (CIs) of dissolution rate from each group, where non-overlapping 95% CIs indicated significant differences between congeners. We conducted this analysis on both our full dataset (full range of Ω_aragonite_) and on a subset corresponding to Ω_aragonite _< 1 (net dissolution expected). We recognize that values for saturation state would be different if we used Ω_calcite_, but have chosen Ω_aragonite_ as a convenient single index to compare dissolution rates, as these indices only differ by the apparent solubility product constant yet are affected similarly by variations in seawater carbonate ion concentration. Additionally, dissolution can be observed in biogenic carbonates when Ω > 1 due to natural elemental and structural heterogeneity [39] and so we focus on the ability of Ω_aragonite_ to primarily reflect changes in carbonate ion concentrations in this experiment.

To determine the contribution of the inner shell layer to dissolution, we conducted bootstrapping analysis on sealed shells (interior coated with silicone waterproof sealant) versus unsealed shells from each species. Finally, to account for slightly variable Ω_aragonite_ between groups, we used linear regression to examine the influence of Ω_aragonite_, mussel species, treatment (sealed or unsealed) and the interaction between treatment and species on dissolution rate after subsetting data to Ω_aragonite _< 1 and log-transforming our dependent variable to account for strong positive skewness. As a sensitivity test, we assessed the model with and without the removal of three high-leverage observations based on hat values and Cook’s distance. Removing these points did not substantially change model coefficients or statistical significance. Diagnostic plots confirmed that our model met assumptions of homoscedasticity and normality of residuals. Results are reported from the full model using all observations.

## 3. Results

**Figure 1.**
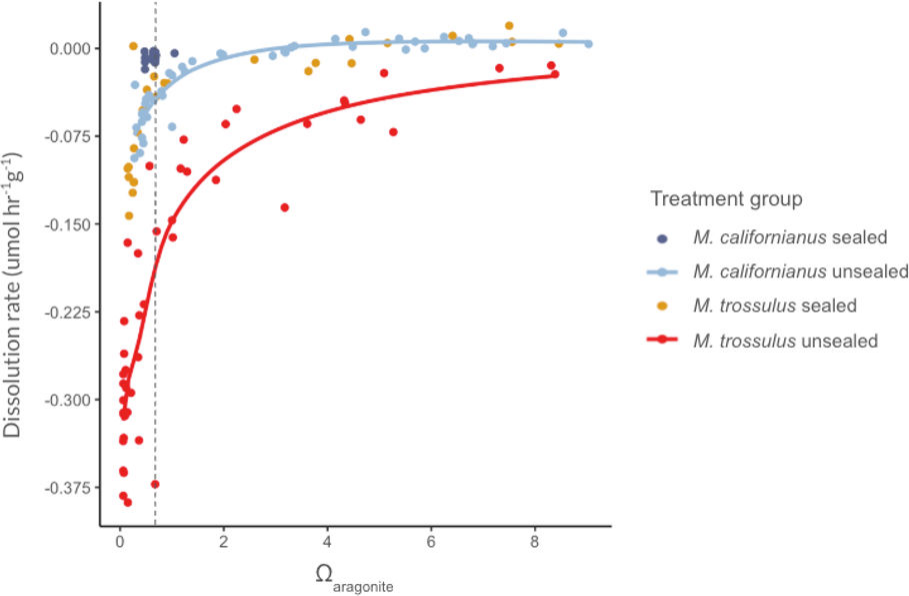
*M. trossulus* dissolution (unsealed shells) exceeds dissolution observed in all other treatments. Abiotic dissolution rate is shown across a range of aragonite saturation state (Ω_aragonite_) for (light blue) unsealed *M. californianus*, (red) unsealed *M. trossulus*, (dark blue) sealed *M. californianus* with an artificially coated interior shell and (yellow) sealed *M. trossulus* with an artificially sealed interior shell. Units reflect normalization of dissolution rate by shell mass. While the x axis represents Ω_aragonite_, the dotted line indicates the point where Ω_calcite_ = 1 for reference. Sealed *M. californianus* were derived from a separate experiment [34] and thus data are not available across a range of Ω_aragonite_.

We refer below to dissolution as the inverse of calcification, and therefore, higher dissolution values represent faster dissolution and negative values represent shell precipitation. For both *M. californianus* and *M. trossulus* shells, abiotic dissolution rate decreased with an increase in Ω_aragonite_ across all treatment groups where Ω_aragonite_ varied (figure 1). There were distinct differences between abiotic dissolution curves of *M. trossulus* and *M. californianus* shells (unsealed), with *M. trossulus* displaying a maximum dissolution rate of 0.39 µmol hr^−1^ g^−1^ at Ω_aragonite_ = 0.15 and *M. californianus* displaying a maximum dissolution rate of 0.09 µmol hr^−1^ g^−1^ at Ω_aragonite_ = 0.28 (table 1). That is, *M. trossulus* abiotic dissolution rate was four times that of *M. californianus* at the lowest target Ω_aragonite_ in our treatments. In addition, while *M. californianus* shells approached an asymptotic calcification rate of approximately 0 µmol hr^−1^ g^−1^ at Ω_aragonite_ > 1, *M. trossulus* shells displayed a calcification rate < 0 across all Ω_aragonite_ for unsealed shells, i.e. no calcification asymptote within the tested Ω_aragonite_ range (maximum Ω_aragonite_ = 9.03).

**Figure 2.**
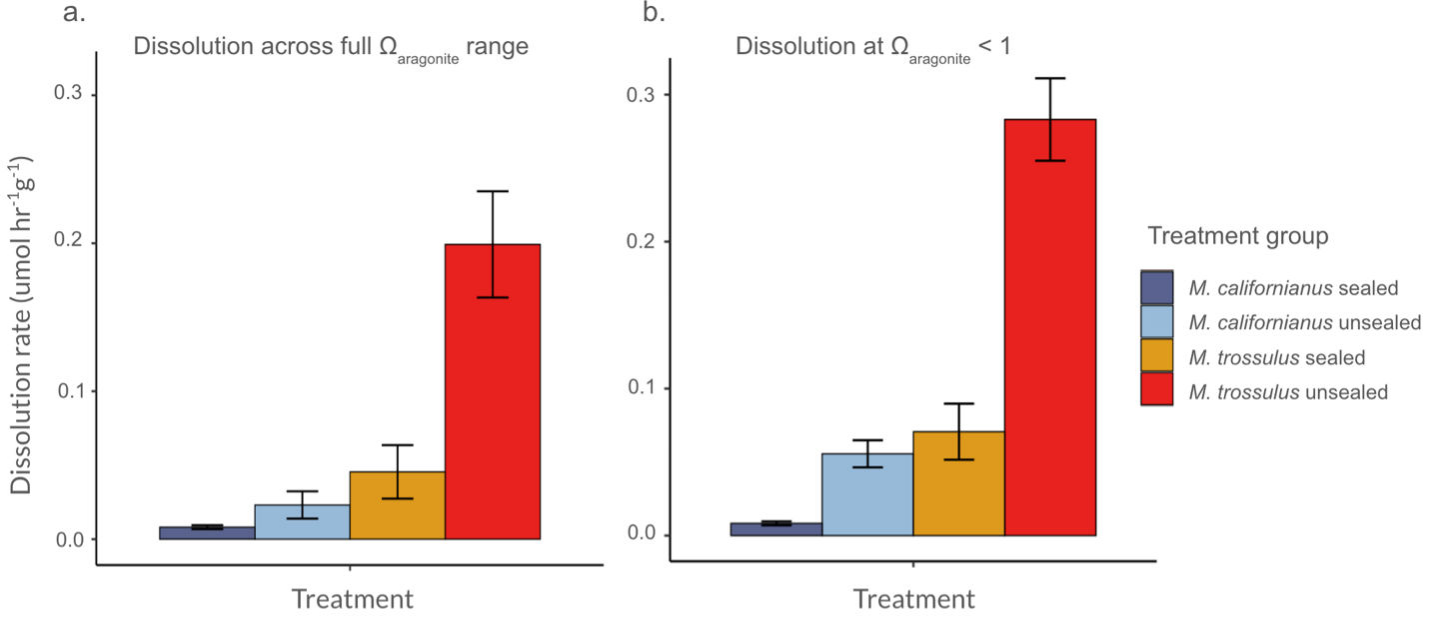
Unsealed *M. trossulus* shells exhibit a significantly and fourfold higher abiotic dissolution rate than *M. californianus*, and a significantly higher dissolution rate than *M. trossulus* with an artificially sealed inner shell. Figure displays mean abiotic dissolution rate (± 95% confidence interval) across treatment groups based on bootstrapping from within-group dissolution rate for (a) shells treated across the full experimental range of aragonite saturation state (Ω_aragonite_) and (b) shells treated with Ω_aragonite_ < 1. Units reflect normalization of dissolution rate by shell mass. Note that for sealed *M. californianus* (dark blue) shells, all but one shell (Ω_aragonite_ = 1.04) were treated with Ω_aragonite_ < 1, and therefore, sealed *M. californianus* data presented in (a) and (b) are nearly identical. (b) allows for inter-group comparison at Ω_aragonite_ < 1.

When comparing dissolution rates across groups at Ω_aragonite_ < 1 , there was a significantly higher dissolution rate in *M. trossulus* (unsealed) than *M. californianus* (unsealed) shells, with non-overlapping CIs of, respectively, 0.28 ± 0.03 and 0.06 ± 0.01 (figure 2b). This finding was consistent when we conducted bootstrapping with sampling from shells across the full range of Ω_aragonite_ versus only shells at Ω_aragonite_ < 1 (figure 2a,b). When comparing shells with an artificially sealed and unsealed interior surface in each species, we found that unsealed *M. trossulus* shells had a significantly higher dissolution rate than sealed *M. trossulus* shells (0.28 ± 0.03 and 0.07 ± 0.02). Likewise, unsealed *M. californianus* shells had a significantly higher dissolution rate than sealed *M. californianus* shells (0.06 ± 0.01 and 0.01 ± 0.00; figure 2b). We note that the constrained range in saturation state for sealed *M. californianus* limits the strength of inference regarding this treatment group. More important is the finding that while *M. trossulus* shells displayed up to 4x higher abiotic dissolution rate than *M. californianus* shells, this effect disappeared when the *M. trossulus* inner shell was artificially sealed.

Model results showed a similar pattern, as species (*M. trossulus* default) showed a significant relationship with abiotic dissolution rate (*β* = 1.86 ± 0.12; *p* < 0.0001; table 2). Treatment (unsealed) also showed a positive and significant relationship with dissolution rate (*β* = 1.76 ± 0.11; *p* < 0.0001). In other words, *M. trossulus* was positively linked to higher dissolution, as was exposure of the interior shell in both species. However, the interaction between treatment and species (unsealed *M. trossulus* default) had a negative relationship with dissolution rate (*β* = −0.64 ± 0.16; *p* < 0.001). In addition, Ω_aragonite_ showed a negative relationship with dissolution rate (*β* = −1.42 ± 0.22; *p* < 0.0001), indicating that dissolution increased at low values of Ω_aragonite_ (table 2).

**Table 2.** Model results: variables correlated with mussel dissolution rate (statistically significant variables in bold).

	*β*	s.e.	*p*
**species (default: *M. trossulus***)	**1.86**	**0.12**	**<0.0001**
**treatment (default: unsealed**)	**1.76**	**0.11**	**<0.0001**
**species × treatment (default: *M. trossulus* × unsealed**)	**−0.64**	**0.16**	**<0.001**
**Ω_aragonite_**	**−1.42**	**0.22**	**<0.0001**
intercept	−3.99	0.15	<0.0001

## 4. Discussion

Two mussel species with overlapping ranges in the California Current System exhibit strongly different abiotic dissolution rates under conditions simulating ocean acidification. *M. trossulus* abiotic dissolution exceeds *M. californianus* dissolution by a factor of four at Ω_aragonite_ approaching 0. This result suggests that *M. trossulus*, a marine mussel that is frequently found within or near freshwater runoff, may be particularly vulnerable to abiotic dissolution while also susceptible to changes in coastal carbonate chemistry associated with freshwater runoff. While net dissolution in nature is also a function of mussel biological control, including compensatory mechanisms that may result from feeding [40], these results provide an early step towards characterizing calcifier susceptibility to ocean acidification in critical coastal habitats.

Abiotic dissolution of *M. trossulus* is significantly higher in the inner shell. After artificially sealing the inner shell of *M. trossulus*, this species showed significantly reduced dissolution relative to unsealed *M. trossulus*. This pattern is consistent in *M. californianus* shells, perhaps because the outer shell is protected from dissolution by the periostracum [27,35] and therefore sealing the inner shell prevents substantial dissolution in both species. There was a negative interaction between *M. trossulus* and treatment (unsealed), suggesting that exposing the inner shell was slightly less deleterious for *M. trossulus* than *M. californianus*. However, this result is uncertain given the limited range of Ω_aragonite_ explored for sealed *M. californianus* shells. In addition, *M. trossulus* exhibited a higher difference in dissolution rate between sealed and unsealed treatments relative to *M. californianus* (difference of 0.21 µmol hr^−1^ g^−1^ for *M. trossulus* and 0.05 µmol hr^−1^ g^−1^ for *M. californianus* between sealed/unsealed treatments at Ω_aragonite_ < 1), indicating that sealing the interior prevented more overall CaCO_3_ loss in *M. trossulus. M. californianus* is the only known *Mytilus* species to contain a separate exterior and interior calcite layer in addition to a third, inner aragonite layer [10], a structure that may lead to less dissolution overall for this species. It is well established that aragonite has a higher solubility than calcite (e.g. [41]), and therefore, the additional calcite layer in *M. californianus* may confer additional protection from dissolution.

*M. trossulus* is typically found in the mouths of rivers or other areas with low or highly variable salinity. For example, *M. trossulus* is common in the Puget Sound and the coastline of the Olympic Peninsula, which receives runoff from the Elwha, Skagit and other large river systems, and represents 70% of animal biomass living on hard sediment in the Baltic Sea, a low salinity system [42]. As ‘wet regions get wetter’ under climate change [43], marine mussels like *M. trossulus* and *M. edulis* that have affinities for lower salinity habitats may be particularly susceptible to reduced calcification given the combined effects of ocean acidification and decreased salinity; alternatively, these organisms may be accustomed to salinity and pH flux (e.g. down-regulating metabolism under suboptimal conditions [40,44]). Salinity generally has a positive relationship with calcification given that low salinity from riverine catchments with typical alkalinity is marked by decreased Ca^2+^ and ${\text{HCO}}_{3}^{-}$ concentration [45]. For example, high-flow systems with high ionic dilution often exhibit low alkalinity [46,47], which Telesca *et al.* [48] suggest is a driver of thin shell structure in *M. trossulus* and *M. edulis* in freshwater-impacted regions [48]. One previous study also showed a negative correlation between salinity and percentage shell aragonite in *M. edulis* in the freshwater-exposed Puget Sound and San Francisco Bay [49]. However, freshwater alkalinity also depends on local lithology; for example, limestone-dominated watersheds and aquifers may generate higher alkalinity in freshwater systems, especially if chemical weathering is stimulated by acid deposition [50,51].

The combination of low salinity and low alkalinity in some freshwater regions may compromise mussel calcification under climate change; however, we speculate that in many cases fresh water provides ample food subsidies to help *M. trossulus* overcome limitations on calcification. Our study measured abiotic dissolution (i.e. it did not account for biogenic plasticity in response to stress). Feeding has been shown to significantly reduce nacre dissolution under high pCO_2_ conditions [27] and there is high biological control over calcification in the inner shell based on the mussel energy budget. Food subsidies may otherwise contribute to compensatory mechanisms under ocean acidification (e.g. increasing protective membranes and accelerating calcium and proton pumps [52]). In previous studies, pH and salinity have had variable effects on the periostracum, suggesting that mussels acclimatize to low pH and salinity in the coastal zone [53].

It is uncertain whether food subsidies are sufficient to compensate for both ocean acidification under climate change and ‘business as usual’ threats to calcification in fresh water-exposed areas (low salinity, low riverine alkalinity), and experiments testing interactive effects of salinity, pCO_2_ and feeding are needed to test realistic portfolios of stress facing nearshore calcifiers. In addition, the ecological implications of compensatory mechanisms under high-food treatments require further study. For example, Telesca *et al.* [48] showed that, as salinity decreased, wild-collected mussels exhibited thinner shells with a thicker periostracum and an increased proportion of calcite and organic matrix, a structure that protects mussels from dissolution but increases vulnerability to predators. *M. californianus* shells had mean dry weights that were three times higher than *M. trossulus* shell weights, indicating thicker shells associated with an additional calcite layer (table 1). If increasing shell thickness occurs through a calcite layer, this might reduce vulnerability to predators in *M. californianus* indicating a compensatory mechanism combating both predation and ocean acidification. Moreover, large size classes of *M. californianus* have demonstrated a positive relationship between seawater temperature and percentage aragonite in shells [49]. Further research is needed to determine how compensating for climate stress (ocean acidification and thermal stress) affects trophic interactions and performance across multiple life history stages and size classes in calcifiers. In addition, policy analyses similar to those initiated in tropical systems (e.g. [54]) are needed to identify local- and regional-scale regulations (e.g. water quality or land-use measures) that may alleviate multiple stressors on freshwater-exposed temperate species under climate change.

## Data Availability

All code and data used in this article is available on Dryad [55].
